# Revisiting the Faure report: Contemporary legacy and challenged legitimacy

**DOI:** 10.1007/s11159-022-09979-1

**Published:** 2022-12-01

**Authors:** Moosung Lee

**Affiliations:** 1 Faculty of Education, University of Canberra, Canberra, ACT Australia; 2 Department of Education, Yonsei University, Seoul, Republic of Korea

**Keywords:** *Learning to be*, Faure report, Lifelong learning, Self-fulfilment, Self-learning, Neoliberal governance, Techno-human co-evolution, UNESCO

## Abstract

Since its publication in 1972, the Faure report has been regarded as a foundational text on the subject of lifelong learning, offering a plethora of ideas and repertoires. This article contemplates why and how the notions of self-fulfilment and self-learning are interrelated and profoundly important in understanding contemporary lifelong learning discourses, and how both have been appropriated by subsequent policy texts embedded in neoliberal thinking. The author argues that pursuing lifelong learning for self-fulfilment becomes voluntary self-exploitation as the individual’s desire to learn unwittingly becomes driven by the instinct to survive and thrive in neoliberal socio-political environments. He also demonstrates that the ideas and repertoires provided in the Faure report function as a fertile ground for lifelong learning discourses, even though the abundant mix of ideas and propositions make it difficult to view the report as an ideologically coherent and conceptually tight-knit blueprint for the future of education. Nonetheless, the author argues that the legacy of the Faure report is still valid beyond its historical specificity. He points out that when read within the context of the unprecedented worldwide experience of COVID-19, the Faure report’s proposition and reservations regarding mass media and cybernetics can shed light on the potential for contemporary technologies to strengthen emancipatory experiences of lifelong learning. Reflecting on this, he suggests that it is necessary to think collectively about how we can appreciate and harness technological innovation as an emancipatory tool to liberate ourselves from ignorance and prejudice through borderless and limitless connections to others, and to learn how to live with them.

## Résumé

Réétudier le rapport Faure : un héritage contemporain et une légitimité remise en question – Depuis sa publication en 1972, le rapport Faure fait figure de texte fondateur sur l’apprentissage tout au long de la vie au sujet duquel il offre pléthore d’idées et de répertoires. Le présent article examine non seulement pourquoi et comment les notions d’épanouissement personnel et d’autoapprentissage sont interdépendantes et profondément essentielles pour comprendre les discours sur l’apprentissage tout au long de la vie, mais aussi comment les textes politiques ultérieurs ancrés dans la pensée néolibérale se les sont appropriées. L’auteur affirme qu’apprendre tout au long de la vie dans une optique d’épanouissement personnel devient une autoexploitation volontaire étant donné que le souhait de la personne d’apprendre incidemment est mu par l’instinct de survie et de réussite dans des environnements sociopolitiques néolibéraux. Il démontre aussi que les idées et répertoires présentés dans le rapport Faure servent de terreau fertile aux discours sur l’apprentissage tout au long de la vie bien que la profusion d’idées et propositions rendent difficile de le considérer pour l’avenir de l’éducation comme un plan cohérent sur le plan idéologique et rigoureux du point de vue conceptuel. Néanmoins, l’auteur affirme que l’héritage du rapport Faure conserve sa validité au-delà de sa spécificité historique. Il indique que lu dans le contexte de la covid-19, une expérience sans précédent dans le monde entier, la proposition et les réserves du rapport Faure concernant les médias de masse et la cybernétique peuvent fournir un éclairage sur ce que les technologies contemporaines sont susceptibles d’apporter pour renforcer les expériences émancipatrices de l’apprentissage tout au long de la vie. En se penchant sur la question, il indique qu’il est nécessaire de réfléchir collectivement à la façon d’apprécier et d’exploiter les innovations technologiques en tant qu’outils émancipateurs pour nous affranchir de l’ignorance et des préjugés en créant des liens sans frontières et illimités avec les autres, et en apprenant comment vivre avec eux.

## Introduction

Since the Second World War, a fast-growing number of international organisations have been established to address various global issues, including global educational development. Among these international bodies, four in particular have played a key role in the field of global educational development, namely the United Nations Educational, Scientific and Cultural Organization (UNESCO); the Organisation for Economic Cooperation and Development (OECD); the European Union (EU), and the World Bank (Lee and Jan 2018). These “big four” have been developing distinctive educational policy discourses since the 1970s, and they have largely maintained their own ideological positioning during the post-2000 period (ibid.).

One aspect of global educational development is lifelong learning. When defining the role of lifelong learning, the OECD’s position has primarily been based on various elements of neoliberal thinking (e.g. economic rationalism, human capital theory, new public management theory, public choice theory, monetarism, vocationalism, etc.), whereas UNESCO’s stance has firmly stood with social democratic liberalism, which values the role of lifelong learning in realising self-fulfilment and humanising society (Lee and Friedrich 2011). The EU has taken a more pragmatic approach to its lifelong learning policy by embracing a broader heterogenous ideological spectrum that can be tailored to the various needs and priorities of EU countries involved (Dehmel 2006; Lee et al. 2008), which is clearly seen in its key policy text, entitled *A Memorandum on Lifelong Learning* (EC 2000). The World Bank joined the policy arena of lifelong learning as a latecomer in the early 2000s. Similar to the EU and the OECD, the World Bank has recognised the importance of various learning contexts and systems (e.g. formal, non-formal and informal), while reframing lifelong learning as a policy tool to rationalise and harness existing learning contexts and systems to meet the challenges of the changing global economy (Lee and Jan 2018).

In more recent years, however, while maintaining their ideological positioning, the big four have also attempted to “patch” some parts of their policy discourses of lifelong learning by accommodating the ideas of their counterparts. For example, the OECD has tweaked its classical concepts of liberalism and human capital into “inclusive” liberalism and “wider” human capital (OECD 2002). Irrespective of whether such adjectives are merely decorative or symbolically substantive labels, they demonstrate that the OECD has reacted to certain criticisms of its lifelong learning discourses through such modifications. In a similar vein, UNESCO’s recent policy texts have acknowledged the role of lifelong learning in adapting to changing labour markets (Lee and Jan 2018). Of course, this kind of “institutional learning” should be seen as “the selective adoption by organisations of characteristics or policies from other organisations” to maintain organisational legitimacy, not as a “wholesale homogenisation” across the global field of lifelong learning policy discourses (Lee et al. 2008, p. 445). Nonetheless, the point here is that there has been a certain degree of mixture of lifelong learning policy discourses across the big four since 2000 (see Lee and Jan 2018 for details).

The origin of such a mixture of lifelong learning policy discourses is not new; it can be traced back to the early 1970s, when UNESCO established the International Commission on the Development of Education, chaired by Edgar Faure. Since the publication of the Commission’s report in 1972 (Faure et al. 1972), the “Faure report”, as it is commonly referred to, has been regarded as a foundational or landmark text on the subject of lifelong learning (Biesta 2021; Field 2001; Medel-Añonuevo et al. 2001; Lee and Friedrich 2011; Elfert 2015, 2018, 2020). One main reason for escalating the status of the Faure report as a legacy text in the policy landscape is that it offers a plethora of ideas and repertoires in relation to lifelong learning discourses. While the abundance of ideas and repertoires in the Faure report may function as fertile ground from which discourses about lifelong learning can emerge, such a mixture of ideas and propositions also makes it difficult to see the report as an ideologically coherent and conceptually tight-knit blueprint for the future of education. In addition, as I detail later in this article, some of the key notions of the Faure report such as self-fulfilment and self-learning have been appropriated far beyond the report’s original intent by proponents of neoliberal thinking. In elaborating this argument, I aim to discuss to what extent the legacy of the Faure report is still valid beyond its historical specificity.

## Self-fulfilment: the continuous process of learning to be a human individual

The idea of lifelong learning is not a modern invention. It can be found in non-Western cultural legacies such as ancient Chinese literature and Islamic letters (Brown 2000). In Western societies, it can be traced back to the work of early 20th-century writers such as Edward Lindeman (1926) in the United States and Basil Yeaxlee (1929) in the United Kingdom. Internationally, UNESCO first started using the term “lifelong education” in its official documents in the 1960s (Lee and Friedrich 2011). René Maheu, UNESCO’s French Director-General during this time, envisioned the idea of *éducation permanente* [literally: permanent education] as a fundamental premise upon which a whole society could be transformed and by way of which its individual members would learn throughout their lives in order to realise personal potential.

In 1971, Maheu asked Edgar Faure, the French minister of education and minister of social affairs at the time, to expand and elaborate the idea of *éducation permanente* as a global vision for the future of education. This suggests that Faure’s understanding of learning throughout life was inherently a French vision and understanding of lifelong education, namely *éducation permanente* (Lee and Friedrich 2011). In this regard, there is some potential for a critique of the Faure report that *éducation permanente* is a Western-centric quasi-political idea about “humanising” educational development (see Finger and Asún 2001).

Re-reading the Faure report today, however, reveals that such a critique is only true on a superficial level. I can certainly see that the Faure report is inherently grounded in European liberalism, given the profound polemics that expound a vision for all individuals’ full realisation of their potential and interests through lifelong education (Lee and Friedrich 2011); “there is no real freedom of choice unless the individual is able to follow any path leading to his [*sic*] goals without being hindered by formalised criteria” (Faure et al. 1972, p. 188).^1^

At the same time, however, it is fair to point out that the Faure report also attempts to go beyond European liberalism, putting aside questions about the success or otherwise of transcending European liberalism. Specifically, the Faure report views self-fulfilment through lifelong education as something that occurs through an individual’s connection with broader society and should contribute to both individual realisation and collective advancement. This is envisaged through the creation of “complete men” in the report:

*through the knowledge it provides of the environment in which it operates[,] education may help society to become aware of its problems and, prodded that efforts are centred on training* “*complete men” who will consciously seek their individual and collective emancipation, it may greatly contribute to changing and humanizing societies* (Faure et al. 1972, p. 56; italics in original).

It should also be remembered that the members of the Commission who contributed to the Faure report (i.e., including Faure’s six co-authors from Chile, Syria, the People’s Republic of the Congo, the Union of Soviet Socialist Republics [USSR], Iran and the United States [US]) not only represent geographical variety but also non-Western ways of knowing. Of course, one may argue that the Commission’s membership configuration does not necessarily mean that all voices and cultural knowledge are equally reflected in the report. Yet the wide range of consultations and documents comprehensively covered by Faure’s International Commission does indicate an effort to go beyond European liberalism (Chapter 1, for example, makes reference to “[t]he African tradition”; ibid., p. 5) in the report’s hightlighting of self-fulfilment through lifelong education.

It is also worth noting that the documents reviewed by the Commission included the radically humanistic ideas proposed by Paulo Freire (e.g. conscientisation), Ivan Illich (e.g. conviviality, de-schooling) (Faure et al. 1972, pp. 20, 21, 75, 139) and the alternative polemics of other public intellectuals at the time (e.g. Paul Goodman) (ibid., p. 5). Of course, caution should be exercised when interpreting the appearance of radical democrats’ ideas within the Faure report, since such ideas are presented only briefly and marginally. It is possible to regard these as mere ornaments inserted to signal some semblance of inclusivity within the context of the whole report. Nonetheless, like those radical democrats, the Faure report is oriented towards a “profound humanism”^2^ which denies any form of educational systems and practices that are authoritarian and oppressive. In other words, the Faure report negates institutions and practices that dehumanise one’s identity as a lifelong learner, given that the essence of lifelong learning presented in the Faure report is about how one can fulfil oneself by learning throughout life, which is an existential process fundamental to all human beings:

Education from now on can no longer be defined in relation to a fixed content which has to be assimilated, but must be conceived of *as a process in the human being*, *who thereby learns to express himself to communicate and to question the world, through his various experiences, and increasingly – all the time – to fulfil himself* (Faure et al. 1972, p. 143; emphases added).

Indeed, this emancipatory feature of the Faure report was well received by key figures inside (or closely related to) UNESCO in the 1970 and 1980s (e.g. Paul Lengrand, Ravindra Dave, Bogdan Suchodolski and Ettore Gelpi) (Wain 2004). These writers, who were so-called maximalists,^3^ were critical of “the authoritarian, uniform, monolithic and unequal design of most education systems in pursuing new pedagogical ideas” (Lee and Friedrich 2011, p. 157; see also Wain 2004). For example, Gelpi (1979), who was in charge of UNESCO’s lifelong education division in the 1970s and 1980s, strongly advocated lifelong education^4^ as a vehicle for emancipation from all kinds of oppression. Resonating with the Faure report, the maximalists acknowledged the important role of schools in building a learning society, which was the main difference from de-schoolers^5^ (Lee and Friedrich 2011). As Kenneth Wain correctly points out, for the maximalists, institutionalisation of all formats of learning *per se* was important in and of itself, but what was more important was “what kind of institutionalisation, and for what purpose” (Wain 2004, p. 39), given the humanistic and emancipatory role of learning in self-fulfilment.

The above excerpt from the Faure report also suggests that lifelong learning is about “the continuing process of making sense of everyday experience” (Jarvis 1992, p. 11). Making sense of everyday experience does not mean a passive process of acknowledging and accommodating what is demanded by external environments. It is more about critically engaging in material and social conditions surrounding an individual, and seeking to challenge such conditions (Kilgore 2001). This process can also be said to be one that causes one to reflect and re-examine one’s ontological position from the social context within which one exists. In this regard, the essence of Faure’s *“Learning to be”* is *a continuous learning process of achieving self-fulfilment*, or, put differently, *it is about learning to be human beings.*

In summary, the Faure report’s notion of self-fulfilment is distinctive from the idea of traditional liberalist self-fulfilment. On the one hand, the Faure report sees self-fulfilment as an individual’s essential right, and on the other hand, the report does not simply view individuals as self-interested agents motivated by a universal egoism. Instead, the Faure report regards self-fulfilment as a continuous learning process of being a human individual in the context of moral obligation and a social contract. As noted above, this perspective is expressed in the concept of “complete man”, who consciously seeks emancipation and therefore supports a greater view of ever-evolving and increasingly humanising societies.

## Self-learning: the modus operandi for self-fulfilment

The main goal of lifelong learning proposed in the Faure report is “self-fulfilment” (Faure et al. 1972, p. 58). This then raises the question how to *realise* self-fulfilment. The answer from the Faure Commission is self-learning. While the report does not provide a formal definition of self-learning, it discusses several principles and pathways:

*Self-learning … has irreplaceable value in any educational system* … Each individual’s aspirations to self-learning must be realized by providing him – not only in school and university but elsewhere too, under conditions and circumstances of all kinds – with the means, tools and incentives for making his personal studies a fruitful activity (ibid., p. 209; italics in original)

The Faure report also states that self-learning should be *assisted* by more flexible and diversified educational paths: “there is no real freedom of choice unless the individual is able to follow any path leading to his goals without being hindered by formalised criteria” (ibid., p. 188). The report further specifies that self-learning should be

assisted by provision of new and varied sources of materials and data, by numerous leisure activities and by social and community programmes likely to promote participation and encourage mutual education (ibid., p. 18).

When explaining the assisted nature of self-learning, the Faure report uses the term “self-directed learning” interchangeably, offering the following definition:

Self-directed learning is not the same as individualised learning; sometimes the learner *chooses* to enrol in a class or group for part of the process. But *the learner himself initiates, selects the experiences* and the persons who *assist him* in learning and *evaluates* the process (ibid., p. 210, emphases added).

Since the publication of the Faure report, the progressive characteristic embedded in self-learning (or self-directed learning) has become an integral part of lifelong learning discourses. In short, self-learning is an important *modus operandi* for self-fulfilment in the Faure report.

## Neoliberal appropriation of self-learning

Notably, the concept of self-learning/self-directed learning has been somewhat twisted in contemporary adult learning discourses. For example, Carmel Borg and Peter Mayo reveal how the idea of self-directed learning permeated into the policy text of a major international organisation by taking the case of EU’s *Memorandum on Lifelong Learning*:

the underlying liberal notions of some of the “old” literature [i.e., UNESCO’s texts] related to lifelong education, bereft, in a number of works, of a collective dimension, with an individualistic emphasis on “self-directed learning”, paved the way for this distortion and neo-liberalisation of the concept, as propounded in the EU *Memorandum* (Borg and Mayo 2005, p. 218).

To further capture the neoliberal appropriation of self-learning specifically, here is another example. Many best-selling self-help books for adult learners feature terminology beginning with “self-” as a prefix (e.g. self-governance, self-mastery, self-development, self-management, self-caring, self-invention, self-renewal, self-making, self-actualisation, etc.) which tends to be translated into neoliberal language (Lee 2017). Drawing on the work of Byung-Chul Han (2015), a German-based contemporary philosopher, I have argued elsewhere (Lee 2017) that, when pursuing self-fulfilment, lifelong learners are encouraged to become entrepreneur selves who are “free and willing” to improve self-value that is exchangeable or commodified as a market value. I further posit that such freedom or free will for pursuing self-fulfilment becomes subordinated to the technology of neoliberal control because it is freedom/free will that paradoxically locks individuals into becoming neoliberal subjects by preventing them from different ways of being, such as aesthetic and democratic orientations. In other words, neoliberal self-fulfilment is a self-hypnosis, motivating individuals to believe that “they can do anything and everything as a free, autonomous, and rational agent” (ibid., p. 146). Paradoxically, this self-hypnosis functions as neoliberal governance where individuals are treated as an improvable asset for a hierarchically ordered global marketplace (Friedrich and Lee 2011).

In line with this neoliberal appropriation, self-fulfilment as one of the main goals of lifelong learning is clearly presented in the OECD’s policy text, entitled *Lifelong Learning for All* (OECD 1996). This report of the “meeting of the Education Committee at ministerial level” in January 1996 defines the goal of lifelong learning as “[individual] creativity, initiative and responsiveness – which contribute to *self-fulfilment*, higher earnings and employment, and to innovation and productivity” (ibid., p. 15; emphasis added). Why the OECD text is characterised as neoliberal becomes clearer especially when comparing it with UNESCO’s Delors report^6^ which was published in the same year.

In a lifelong learning approach, it is the role of government to promote the development of appropriate “bridges” and “ladders”… in which the various elements of education and training provision can be articulated (OECD 1996, p. 184).

[A]n education system must operate within the context of a social compact …. Governments have a huge responsibility to act as the brokers of this compact (Delors et al. 1996, p. 223).

As pointed out elsewhere (Lee and Friedrich 2011), these two ostensibly similar passages convey profoundly different messages. UNESCO’s Delors report, revitalising the main thrust of the Faure report (Lee 2007) in promoting the operation of a lifelong education system, designates governments as the key agents who themselves “have a huge responsibility” for actively providing and arranging resources and opportunities for individual learners.

In the OECD’s report, however, governments are merely “promoters” who cheer on other educational agencies to be “bridges” and “ladders” for providing education and training. In other words, governments are not supposed to take on the direct role of being bridges and ladders by themselves, but are instead defined as promoters and cheerleaders in the project of building a lifelong learning system. This reduced role for governments in education echoes neoliberal governance. That is, the OECD’s neoliberal governance highlights “the optimal development of human capital through an investment in lifelong learning strategies” in which the sources of investment are individual learners or private sectors (Wain 2000, p. 39).

In summary, the “self-learning” proposed by the Faure report as an integral part of lifelong learning has been appropriated by the OECD’s neoliberal governance. Individual learners are encouraged to learn throughout their life, but their freedom/free will to learn is only legitimised when reduced to exchangeable, commodifiable and improvable assets for a hierarchically ordered global marketplace. For the OECD, self-learning is seen as a central catalyst for knowledge production in a learning economy where the goal of an individual’s learning is to serve the creation of “new products, new techniques, new forms of organisation and new markets” (Lundvall 1992, p. 8). By extension, within this neoliberal governance, the failure to learn (i.e., failure to be a competitive and flexible member of a country’s workforce) becomes a problem that can be entirely attributed to the individual’s responsibility.^7^

## Bringing lifelong learning back from deceptive self-caring

As noted above, within this neoliberal context, lifelong learners are cheered on in their self-learning to pursue self-fulfilment in a manner which is narrowly reduced to improving individuals’ competitiveness and employability, and which moreover suggests that this is something that the individual should be responsible for (Lee and Friedrich 2011). In a similar vein, Gert Biesta captures this prevailing phenomenon in the contemporary discourses of lifelong learning as: “from learning to be, to learning to be productive and employable” (Biesta 2021, p. 6). To articulate such a shift, Biesta contrasts “lifelong education as a right” with “lifelong learning as a duty” (ibid., p. 10). He goes on to argue that “the *emancipation of education itself* may be a more meaningful way forward, that is, one in which there may still be [a] future for Faure’s legacy” (ibid., p. 3, emphasis added). This proposition is paralleled with a body of literature concerning the terminological shift from “lifelong education” to “lifelong learning” (e.g. Boshier 1998; Jakobi 2009; Lee et al. 2008; Nemeth 2015).

As a matter of fact, the two terms were used interchangeably from the 1960s to the 1980s in order to designate the concept of “learning throughout life”. Since the 1990s, however, the term “lifelong learning” has been accorded preference by the major international bodies in their policy texts (Lee and Jan 2018). Like Biesta’s argument, the terminological shift seems to frame what can be thought and said about “learning throughout life” given the different underlying motivations in choosing the preferred or official term for each international organisation. Roger Boshier (1998) was one of the earliest scholars to unpack the policy implications of the terminological shift from lifelong education to lifelong learning, arguing that it was a turn towards neoliberal vocationalism of lifelong education. Looking into the historical evolution of “learning throughout life” from the Faure report to the mid-1990s, Boshier’s critique makes a valid point.

At the same time, however, I have reservations about drawing a sharp line between the two terms. Although understanding that a neoliberal appropriation of the concept of “learning throughout life” exists, the sharp demarcation between the two terms risks making “lifelong learning” a purely neoliberalist phenomenon, which it is not. In other words, overstating a difference between the two terms bears the risk of blurring or even losing the Faure report’s original intention which highlighted the term, “learning” as a core precept of the report, as an existential and reflective human experience. Therefore, rather than treating the two terms as if they were placed on ideologically opposing vectors, resurrecting the term “lifelong learning” as a fact of our lives that can lead us into aesthetic-democratic being, where the self does not always grow in a self-interested way (Friedrich and Lee 2011), is a more meaningful way forward.^8^

In line with this, I acknowledge, on the one hand, Biesta’s concern about “lifelong learning as a duty” given that the neoliberal subject is an enslaved self (McGee 2005). His/her lifelong learning is a never-ending duty required for upgrading work-related skills or vocational qualifications to remain employable in a globally hierarchical market place (Biesta 2021; Lee and Friedrich 2011). On the other hand, it should be noted that neoliberal governance in late capitalist societies defines the individual’s relentless efforts and responsibilities as solutions in addressing systemic problems (Türken et al. 2016). Under this kind of neoliberal social system, “lifelong learning as a duty” can ironically be perceived by the enslaved self as a valuable opportunity, given that when a duty is pursued “voluntarily” and “willingly” as the only way to look after oneself, it is no longer a simple duty in nature. The duty becomes “deceitful, fallacious, self-caring” in the sense that it merely serves the purpose of neoliberal governance. Putting it differently, the neoliberal *subject* views lifelong learning not as a duty but as a *project* by which they believe they can survive and thrive. In this regard, neoliberal lifelong learning is self-exploiting. In his book entitled *24/7: Late Capitalism and the Ends of Sleep*, Jonathan Crary’s warning resonates with the self-exploited nature of lifelong learning in neoliberal governance:

Self-fashioning is the work we are all given, and we dutifully comply with the prescription continually to reinvent ourselves and manage our intricate identities. As Zygmunt Bauman has intimated, we may not grasp that to decline this endless work is not an option (Crary 2013, p. 72).

Fifty years after the publication of the Faure report, we find ourselves in a treacherous dual situation. On the one hand, we believe that we have free will to pursue lifelong learning. On the other hand, pursuing lifelong learning for self-fulfilment requires what might be termed “voluntary self-exploitation”, especially when we desire to survive and thrive in neoliberal socio-political environments. Under neoliberal governance, we are proactively and voluntarily subject to forms of external dictation or measures, ultimately heading towards physical and psychological burnout (Han 2015; Lee 2017; McGee 2005). The Faure Commission certainly never intended and could never have imagined the deceptive new undertone to the term *lifelong learners* orchestrated and held captive by neoliberal interests.

## In defence of the Faure report

The gloomy situation described above may lead us to questions like, What is still valid in the Faure report?, What can be learned from the report?, and What is the legacy of the report? A number of studies (e.g. Biesta 2012, 2021; Elfert 2015, 2018, 2020; Field 2001; Lee and Friedrich 2011) have already addressed these questions in detail. Among the ideas, directions or implications they have identified as the legacies of the Faure report are: a humanistic and utopian vision (Elfert 2015, 2018, 2020); lifelong education for emancipation (Biesta 2021); solidarity through lifelong education (ibid.); re-centring the focus of education from schooling to non-formal and informal education (Field 2001); the vision and notion of the learning society (Biesta 2021; Elfert 2018); etc.

The 1996 Delors report also states that “[the Faure report’s] recommendations are still very relevant [for] the twenty-first century” (Delors et al. 1996, p. 21). By proposing the “four pillars” of education, “*learning to live together”, “learning to know”, “learning to do”*, and *“learning to be”* (ibid., pp. 20–21; italics in original), the Delors report aimed to elaborate the Faure Commission’s vision of a learning society as “the necessary utopia” (Lee and Friedrich 2011). In addition, the present article complements earlier studies by focusing on self-fulfilment and self-learning as key ideas highlighted by the Faure report. It seems that all these elements of the Faure report’s legacy have enjoyed an extended shelf life for innovative and democratic educational ideas.

Despite the fact that the legacy of the Faure report is relatively well-documented, there is another element of this legacy which has rarely been addressed in the literature, namely the role of science and technology in lifelong learning and, more broadly, techno-human co-evolution. As identified by the research literature, the Faure report is often characterised as idealistic, utopian and humanistic, as connoted in its title *Learning to Be*. As Maren Elfert points out,

The title *Learning to be* reveals the influence of existentialism on the report that placed the focus on the human condition and on the role of education for the development of every individual’s potential (Elfert 2020, p. 19).

At the same time, the Faure report takes a “pragmatic” (ibid.) approach to proposing action plans or educational planning with a focus on technological advancement and its significant relationship with individual learners and social progress. In the report’s preamble, in the section labelled “The instruments of change”, Faure himself highlights that:

*The commission accordingly underlined the fact that despite doubts and differing orientations, and whatever the progress or saving which might be obtained from certain changes in the traditional educational system, the very heavy demand for education due on the one hand to the gradual prolongation of school-attendance to optimal age, and, on the other hand, to the institution of a genuine lifelong education, can only be met if instruments derived from modern technology, with its limitless possibilities, are put to use on an adequate scale and with appropriate means* (Faure et al. 1972, p. xxxvi; italics in original).

Earlier in the preamble, in the section labelled “The scientific and technological revolution: education and democracy”, Faure emphasises that

the scientific-technological revolution has simultaneously conquered the mental world, with its immediate transmission of information over any distance and its invention of increasingly perfected, rationalized, calculating machines. This is a phenomenon which necessarily affects all of humanity (ibid., p. xxiii).

Amid technological advancements, the Faure report views mass media and cybernetics as having the great potential to build a learning society.

[T]he revolution in mass media and cybernetics affects everyone everywhere … The scientific-technological revolution therefore places problems of knowledge and training in an entirely new light, giving man entirely new possibilities of thought and action; and, for the first time, it is truly universal (ibid., p. xxiii).

Acknowledging the important role of mass media (radio and television) and cybernetics in education, the Faure report describes mass media and cybernetics as “the instruments of change” (ibid., p. xxxiv), given their emancipatory and up-scalable nature. The report posits that:

radio and television are put to use outside and parallel to education strictly speaking …. It is commonly felt that computerized data processing should be restricted to higher studies; yet, on the contrary, it is most important to plan to give very young children some introduction to the elementary language of machines. First, because algorithms correspond to a remarkable logical method. Second, because contact with this “mysterious” power often greatly strengthens motivation towards knowledge (Faure et al. 1972, p. xxxiv).

Some critics may view the Faure Commission’s advocacy of science and technology as a techno-capitalist way of extending traditional schooling into a whole society. For example, Martin Carnoy (1974) as a member of the US Commission for UNESCO reviewed the Faure report and provided several critiques. One of the main criticisms was about the Faure Commission’s naïve view of the role of science and technology in a learning society, given that the Faure report treats science and technology as a “neutral” input for the provision of the learning society. Furthermore, Carnoy points out that the Faure report overlooks a critical question of “*who* will begin the learning society and *who* will control the science and technology which is used to produce that learning society” (Carnoy 1974, p. 57; emphases in original). It seems true that the Faure report does not explicitly discuss the question of *who*, but primarily focuses on answering the question of *how* science and technology can play out in the formation of the learning society. Additionally, in my view, the Faure report’s depiction of how mass media, cybernetics and algorithms can be pivotal for the success of a learning society is largely futuristic, but also technologically rationalist:

Cybernetic pedagogy … operates at the level of self-regulating individual micro-systems such as adaptable teaching machines, and also among macro-systems such as institutions confronting an infinite variety of individual differences among pupils (Faure et al. 1972, p. 115).

*They* [ecological and cybernetic models] *are sturdy, self-adjusting, self-balancing and self-renovating. Thus, we have reached the point where we foresee methods of organizing education which are based on the principles of dialogue between man and machine providing the possibility for wide-spread individualized learning* (ibid., p. 143; italics in original).

While the Faure report stresses the role of science and technology in building the learning society, it is equally true that it expresses caveats and concerns for the technologisation of human beings. Elfert (2022) provides insights into the latter:

… the existentialist-Marxist concept of “alienation” appears 10 times in the Faure report. Most of these instances refer to technical progress that the report considered to be a double-edged sword that has the potential to bring about positive change, but can also be “a source of iniquity, alienation and new tyrannies” (p. 101). The report expressed concern about “obsessive forms of propaganda” [p. xxiv] of “mass-communication media” [p. 191], “behavioural conformity which may be imposed on [human beings] from the outside” [p. xxiv], and “increasing possibilities for influencing human behaviour” (p. 102) (Elfert 2022, n.p., quoting from the Faure report).

The above caveats are aligned with the Faure report’s humanistic and democratic vision of learning throughout life. At the same time, such concerns in the Faure report can be seen as contradictory to its stress on science and technology in the learning society. I speculate that this discursive dissonance within the report may be attributed to the pragmatic approach taken by the Faure Commission. Elfert (2020) argues that the Faure Commission’s view of lifelong education “blended the post-war idealism with the critical spirit of the 1960s and the humanistic-cosmopolitan worldview that permeated the report”, and it co-existed “in tension with human capital theory, which around the same time fuelled what was seen as a more pragmatic approach to educational planning” (Elfert 2020, p. 19). According to Elfert, under these conflicting circumstances, the Faure Commission undertook “a delicate balancing act and a pragmatic intervention” (Biesta 2021, p. 4, referring to Elfert 2018).

In a similar vein, Carnoy’s critique of the Faure report below shows how the Faure Commission was playing a delicate balancing game to please various stakeholders. In his critique, Carnoy summarises four main views of school development which evolved over time, namely evolutionary idealism, the pluralist view, the human capital view, and the structural functionalist view (Carnoy 1974). He then points out that the Faure report “at one time or another chooses to interpret the role of schooling from all these views. Yet, all of the views have conflicting elements and some of them conflict in fundamental interpretation” (ibid., p. 55). In short, it may have been a daunting task for the Faure Commission to create a policy text that could be an ideologically coherent and conceptually tight-knit blueprint for the future of education. That is, the Faure report’s stance on the role of science and technology in lifelong education can be regarded as an (inevitable) repercussion of the Faure Commission’s balancing act.

More recently, another defence for the Faure report’s promotion of the potential of mass media and cybernetics emerges from the unprecedented, worldwide experience of COVID-19. Since early 2020, the pandemic has created extraordinary challenges for our everyday life, including education and learning activities. The pandemic has revealed the limits of a conventional model of education systems across many countries (Reimers 2022). At the same time, paradoxically, the COVID-19 crisis has highlighted the potential of contemporary online media (e.g. learning management systems, videoconferencing platforms like Zoom, cloud-based team collaboration platforms like Microsoft Teams, etc.) in coping with disruptions caused by the pandemic. Although there are still issues and challenges in relation to using contemporary online media (e.g. accessibility, surveillance, commercialisation), the pandemic is teaching us that there is a role that contemporary online media can play for “learning throughout life”. In this regard, the Faure report’s proposition of the potential of mass media is still valid, on the condition that we are equipped with cautionary, critical approaches.

Finally, as far as educational change in the future is concerned, the Faure report’s emphasis on cybernetics and algorithms in the learning society may still have a valid point. To support this point, recent artificial intelligence (AI) technology development led to the following phenomenon. In March 2016, “AlphaGo”, the AI-based computer programme of the complex board game Go, defeated Lee Sedol, the (almost) invincible world champion. This “AlphaGo shock” clearly tells us two things: (1) a machine can outperform humans in complex tasks requiring high-order thinking abilities such as problem-solving and creativity; and (2) in the future, we and next-generation learners will have to live with technological advancements such as AlphaGo in our daily life. This means that next-generation learners

will have to learn about how to live together with other human beings and how to be as a human-being amid such unprecedented, overwhelming technological advancements that would substantially change the way we relate to others (Lee 2020, p. 5).

I do not think that we can simply deny all technological advancements that fundamentally change how we form relationships with others from different socio-cultural backgrounds. Rather than dismissing all kinds of technological innovation simply as cyber-capitalism, which is just a wholesale criticism, what we need is to collectively think about how to live together with other human beings amid ever-advancing technologies.

Revisiting the Faure report fifty years after its publication is useful because it allows us to think collectively about how we can appreciate and harness technology as an emancipatory tool to liberate ourselves from ignorance and prejudice through borderless connections, and to learn how to live with others. I believe that this is one of the covert messages conveyed in the Faure report when it proposes the role of science and technology in a learning society. Perhaps we are on the verge of *l’heure entre chien et loup* – literally, “the hour between dog and wolf”. There may be some ambiguous hours ahead (either dawn or dusk) during which we cannot tell whether contemporary technological advancement is a dog or a wolf for our learning throughout life.

## Footnotes

1 When it talks about individuals and learners, the Faure report refers only to “man”, using “he” and “him” throughout. While this is offensive to today’s readers, it was still common usage when the report was being drafted.

2 The term “profound humanism” is borrowed from John Field, who argues that UNESCO’s lifelong education, including the Faure report, “was surely subordinated to a profound humanism” during the 1970s (Field 2001, p. 13).

3 In this context, *maximalists* were those who promoted “a fundamental transformation of society” (Cropley 1979, p. 105, quoted by Elfert 2016, p. 147).

4 Gelpi continuously used the term “lifelong education” instead of lifelong learning to avoid any pitfalls of neoliberal discourse.

5 *De-schoolers* held the opinion that inadequate institutionalisation or over-institutionalisation of education systems with governments’ centralised control and beaurcatic accreditation of learning experiences and opportunties contributes to dehumanisation.

6 In 1993, UNESCO set up the International Commission on Education for the Twenty-First Century and appointed Jacques Delors, then President of the European Commission, as its chairman. This Commission’s report, entitled *Learning: The treasure within* (Delors et al. 1996), is commonly referred to as “the Delors report”.

7 See English and Mayo (2021) for more updated critiques of neoliberal entrenchment in contemporary lifelong learning.

8 For these reasons, I use the two terms interchangeably here, albeit being aware of the implications of the terminological shift (see also Field 2001; Tuijnman and Boström 2002).
